# Novel recombinant papillomavirus genomes expressing selectable genes

**DOI:** 10.1038/srep37782

**Published:** 2016-11-28

**Authors:** Koenraad Van Doorslaer, Samuel Porter, Caleb McKinney, Wesley H. Stepp, Alison A. McBride

**Affiliations:** 1 Lab of Viral Diseases, NIAID, NIH, Bethesda, MD, USA

## Abstract

Papillomaviruses infect and replicate in keratinocytes, but viral proteins are initially expressed at low levels and there is no effective and quantitative method to determine the efficiency of infection on a cell-to-cell basis. Here we describe human papillomavirus (HPV) genomes that express marker proteins (antibiotic resistance genes and Green Fluorescent Protein), and can be used to elucidate early stages in HPV infection of primary keratinocytes. To generate these recombinant genomes, the late region of the oncogenic HPV18 genome was replaced by CpG free marker genes. Insertion of these exogenous genes did not affect early replication, and had only minimal effects on early viral transcription. When introduced into primary keratinocytes, the recombinant marker genomes gave rise to drug-resistant keratinocyte colonies and cell lines, which maintained the extrachromosomal recombinant genome long-term. Furthermore, the HPV18 “marker” genomes could be packaged into viral particles (quasivirions) and used to infect primary human keratinocytes in culture. This resulted in the outgrowth of drug-resistant keratinocyte colonies containing replicating HPV18 genomes. In summary, we describe HPV18 marker genomes that can be used to quantitatively investigate many aspects of the viral life cycle.

Worldwide, human papillomavirus (HPV) infections are one of the most common sexually transmitted infections. While most infections are asymptomatic, persistent infection with certain HPV types has been shown to be the causative agent of an increasing number of human cancers^1^. All known papillomaviruses have a small dsDNA circular genome of approximately 8 kb that encodes for six to eight genes. Because of this limited coding capacity, the HPV life cycle is strictly dependent on host factors and is tightly associated with keratinocyte differentiation. Following infection, the viral genome must amplify to a low copy number and become established as a stable, extra-chromosomal replicating element. This initial phase is followed by the maintenance phase, during which viral genomes are maintained at a constant copy number in actively dividing cells. Finally, terminal differentiation of the infected cell triggers high-level viral DNA amplification and capsid protein production resulting in the assembly of infectious virus. Because of this dependence on cellular differentiation, the viral life cycle cannot be studied using standard, monolayer cell culture systems.

Early studies showed that viral particles isolated from warts were used to study the ability of the viral genome to be maintained^2^. However, similar samples are hard to obtain and the observation that transfected human papillomavirus genomes could establish persistent replication in human primary foreskin keratinocytes was a major breakthrough^3^. This transfection-based system has helped elucidate many events in the viral life cycle^3^. Using this method, it has been shown that oncogenic HPV types will replicate in primary human keratinocytes and provide these cells with a growth advantage, eventually leading to their immortalization^4^,^5^. A correlation has been observed between the oncogenic potential of HPV types and their ability to score in a quantitative colony forming assay^6^. Traditionally, the viral DNA is co-transfected with a plasmid encoding a selectable marker. However, since the selectable gene is supplied in trans from a non-replicating plasmid, selection can only be maintained while the non-replicating plasmid is present in the cell (typically a few days). Furthermore, this can result in false positive (cells transiently resistant to the selectable marker but no HPV18) and false negative (cells containing HPV18 but not resistant to the selectable marker) results^6^. More recently, HPV genomes packaged in viral particles (known as quasiviruses)^7^ have been used to investigate early events in the viral life cycle^8^,^9^,^10^. However, HPV-derived quasiviruses can only contain a single plasmid or genome^7^.

While studying HPV genome persistence, it was noted that fragments of the late region were not required for long term replication^11^. This observation led to the hypothesis that the late region of the viral genome could be replaced by selectable markers, thus creating viral genomes carrying a selectable marker in cis. The current study provides evidence that insertion of these selectable markers into the viral genome does not affect the ability of these genomes to replicate and to transcribe viral genes. Furthermore, the presence of the selectable markers in cis significantly improves the robustness of the quantitative colony formation assay. Finally, quasiviruses produced from packaging these viral genomes into viral capsids can infect primary human keratinocytes, providing a biologically relevant, and quantitative, colony forming readout of viral infection and establishment. These marker genomes represent a powerful new tool for screening early stage infections *in vitro* to study key processes in HPV establishment.

## Results and Discussion

**Figure 1:**
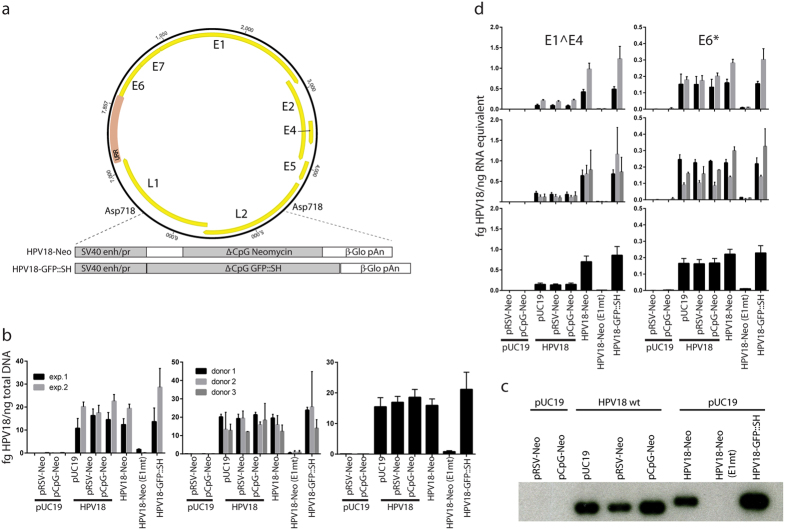
Insertion of marker genes does not affect the ability of the viral genome to replicate short-term. (**a**) Recombinant HPV18-based genomes were generated to contain expression cassettes for selectable marker genes. A 1479 bp portion of the HPV18 L2 and L1 genes was replaced by the expression cassettes as described in Methods. (**b**) DNA was harvested from HFKs 96 hours post-electroporation. Prior to analysis, total cellular DNA was digested with NcoI and DpnI to linearize the genome and remove unreplicated input DNA. Viral DNA was detected by DNA qPCR. Data shown was normalized by cellular β-actin levels. The data are the averages of two independent experiments, each with three different isolates of keratinocytes. Data is grouped by experiment (left; n = 2), by isolate (middle; n = 3), and compiled (right; n = 6). Error bars indicate the standard error of the mean (SEM). (**c**) Cellular DNA was analyzed using Southern blot with a [^32^P]-labeled HPV18 DNA probe. (**d**) Total cellular RNA was harvested from cells 96 hours post electroporation. Viral HPV18 E1^E4 and E6*I cDNA was quantified using qPCR. Data shown was normalized by cellular TBP levels. The data are the averages of two independent experiments, each with three different isolates of keratinocytes. Data is grouped by experiment (top; n = 2), by donor (middle; n = 3), and compiled (bottom; n = 6). Error bars indicate the standard error of the mean (SEM).

It was previously noted that a sub-genomic replicon, with a substantial part of the late region of HPV18 deleted, replicated efficiently when transfected into primary HFKs^11^. In the present study, a similar 1479 bp fragment (nt. 4795-6273 in HPV18) was replaced with different transgenes to provide resistance to drug selection in mammalian cells (Fig. 1a). These transgenes encoded either an aminoglycoside phosphotransferase gene that confers resistance to neomycin, or a GFP::SH fusion gene that encodes a red-shifted variant of the jellyfish green fluorescent protein (GFP) fused to the Sh-ble gene that confers resistance to Zeocin. To minimize the potential deleterious effects of inserting a transgene into the HPV genome, the selectable markers were based on artificial genes optimized for long-term *in vivo* expression^12^. The HPV18-Neo virus contains a Neo gene which is completely devoid of CpG dinucleotides. This modified Neo gene confers resistance to G418 in mammalian cells. Likewise, the GFP::Sh gene has been modified to be devoid of CpG dinucleotides. Expression from these synthetic genes is driven by a modified, CpG-free SV40 promoter. Long-term expression of (virally transduced) transgenes is an active field of study^12^,^13^, but it has been shown that CpG motifs located within the promoter and/or open reading frame (ORF) are associated with a loss of transgene expression^14^,^15^. Furthermore, the HPV18 genome has a decreased CpG content^16^, suggesting that CpG motifs may affect the fitness of these viruses^17^.

Upon transfection into cells, HPV18 genomes will undergo initial replication and express early viral genes^9^. To ensure that insertion of non-viral transgenes did not affect these initial steps of the viral life cycle, primary keratinocytes were electroporated with wild-type or HPV18 derived marker genomes and viral replication and transcription was assessed. Four days post-transfection, total RNA and whole-cell DNA was extracted. Following DpnI digestion to eliminate unreplicated viral DNA, replicated viral DNA was measured by quantitative PCR (qPCR; Fig. 1b) and Southern blot analysis (Fig. 1c). As a negative control, a viral genome unable to replicate (E1 mt) was included. Notably, insertion of marker genes did not affect the ability of the viral genomes to replicate. Analysis of viral transcription by quantitative, RT-PCR indicated that transgene insertion slightly increased E1^E4 transcription; however, the levels of E6*I, a true early transcript, were unaltered (Fig. 1d).

In the experiments shown in Fig. 1, the wild type genome was co-transfected with plasmids providing neomycin resistance, although drug selection was not applied in this short term experiment (duplicate plates were used in long-term assays with drug selection). pRSV-Neo contains a wild type, CpG containing Neo gene driven by an RSV promoter. The pCpG-Neo plasmid contains the modified CpG free gene driven by the same SV40/I-E2CK promoter as present in the HPV18-Neo construct.

**Figure 2:**
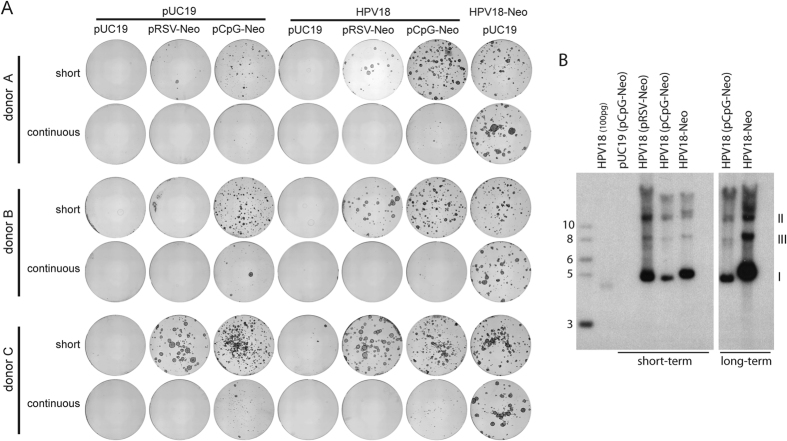
Presence of the Neomycin resistance gene in the viral backbone increases the robustness of colony formation. 1 × 10^6^ cells were electroporated with 1 μg of each indicated plasmid. 2 × 10^5^ cells were plated on 10 cm plates. One-day post-electroporation cells were treated with G418 as indicated. Cells were cultured for approximately 2 weeks. (**A**) Keratinocyte colonies were stained with methylene blue. (**B**) DNA was harvested from colonies obtained following continuous selection. Viral DNA was detected by Southern blot analysis with a [^32^P]-labeled HPV18 DNA probe.

During the viral life cycle, the initial amplification stage is followed by long-term persistence of the viral genome. To test the ability of HPV18 derived marker genomes to be maintained long term, three different isolates of primary HFKs (from different donors) were transfected with HPV18 or HPV18-Neo genomes, and analyzed for their ability to form drug-resistant colonies of keratinocytes after selection. The wild type genome was co-transfected with the pRSV-Neo and pCpG-Neo plasmids providing neomycin resistance (Fig. 2). Two distinct selection schemes were tested: cells were selected with 400 μg/ml G418 for six days, followed by an additional two week incubation period. During this incubation, cells were either left untreated (short term selection), or selected with 200 μg/ml G418 (continuous selection). While short term selection was sufficient for one out of three tested donors (Fig. 2A donor A; compare pCpG-Neo with or without the HPV18 wt genome), it did not allow a clear differentiation between HPV18 positive and negative colonies in the other two donor lines (Fig. 2A donor B-C; compare pCpG-Neo with or without HPV18 wt genome). This donor dependence has been previously described^6^ and can limit the usability and reproducibility of the experimental read-out in the colony forming assay. However, the presence of the Neo resistance gene in cis should allow long-term selection of cells that maintain the viral genome extra-chromosomally. Indeed, the ability to maintain continual selective pressure results in a more robust colony formation that is independent of the host genetic background (Fig. 2A). Importantly, as shown by southern blot analysis of viral DNA, G418 selected colonies maintain the viral genome extra-chromosomally (Fig. 2B). Notably, cells transfected with only the pCpG-Neo selectable marker-containing plasmid formed a few, small colonies even after long term selection, most likely due to integration of the selectable marker plasmid into the host DNA. However, under the same conditions, co-transfected HPV18 DNA is maintained as an extrachromosomal element (Fig. 2B), indicating that long-term neomycin selection does not favor integration of the viral genome.

The increased robustness of the quantitative colony formation assay can likely be explained by the fact that, as the cells divide, the co-transfected selectable marker is lost over time, thereby limiting how long co-transfected cells can be selected. However, cells that survived this initial selection period will form robust colonies, even if the viral genome does not establish and is lost. Therefore, individual colonies need to be tested to ensure the presence of viral genomes^6^. This issue is exacerbated by the fact that the pCpG-Neo plasmid was optimized for long-term maintenance and expression in mammalian cells^18^. These issues can be avoided by including the transgene in cis to the viral genome, thereby allowing long term selection and dramatically improving the robustness of the colony formation assay.

**Figure 3:**
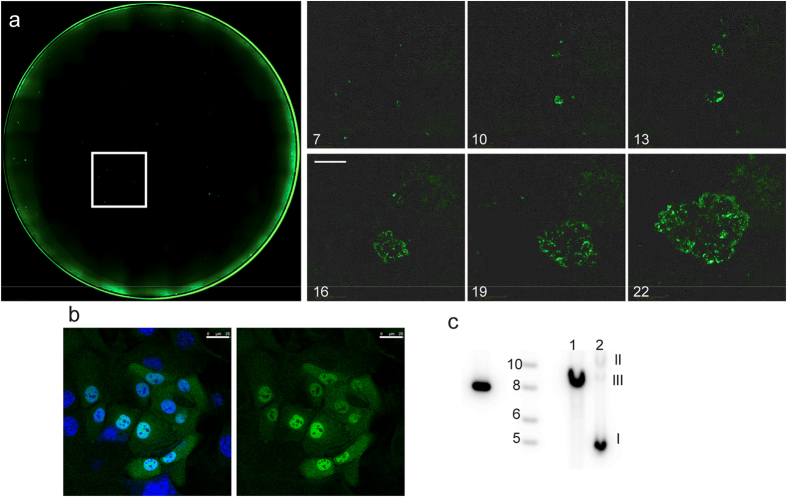
HPV18 positive cells can be visualized using live cell microscopy. (**a**) Primary foreskin keratinocytes were electroporated with 2 μg HPV18-GFP::SH. 85,000 cells were plated in a well of a 12 well plate. 24 hours post transfection, cells were selected with 25 μg/ml zeocin. Selection was maintained for 96 hours, after which the selection was lowered to 2.5 μg/ml. An Incucyte live cell imager was used to image the entire well. Numbers indicate days post transfection. The scale bar equals 1.3 mm. (**b**) Primary foreskin keratinocytes were transfected with 2 μg HPV18-GFP::SH. 1 × 10^6^ cells were plated on a 10 cm cell culture plate and selected as in (**a**). Colonies were pooled onto coverslips. Cells were fixed and GFP levels were detected using standard immunofluorescence methods. The image shows a keratinocyte colony surrounded by irradiated fibroblasts. The scale bar equals 25 μm. (**c**) Viral DNA was detected by Southern blot analysis with a [^32^P]-labeled HPV18 DNA probe. DNA in lane 1 was digested with an enzyme that linearizes HPV18-GFP::SH, while the DNA in lane 2 was left untreated. The three topological forms of DNA (supercoiled, nicked and linear) are indicated on the right.

The choice of selectable markers can dramatically impact cell line development^19^. For example, in certain cell lines less than 50% of G418 selected cells indeed express the transgene^19^. However, Zeocin-resistant cell populations have been described to exhibit better transgene stability in the absence of selection pressure compared to other selection agents^19^. Importantly for our studies, NIH-3T3 J2 fibroblasts are resistant to Zeocin at concentrations lethal to primary HFKs (unpublished observations). HPV18 marker genomes containing the GFP::Sh gene were transfected into cells, which were selected with Zeocin until colonies were formed. Live cell microscopy was used to follow colony formation in real time (Fig. 3a; supplemental movie 1). This analysis showed that while several cells initially express GFP, the recombinant genome only becomes established in a small percentage of cells that give rise to Zeocin-resistant, GFP expressing colonies. Most cells in each colony express primarily nuclear GFP (Fig. 3b). The HPV18-GFP::SH recombinant genomes establish colonies that maintain the viral genome extra-chromosomally (Fig. 3d).

**Figure 4:**
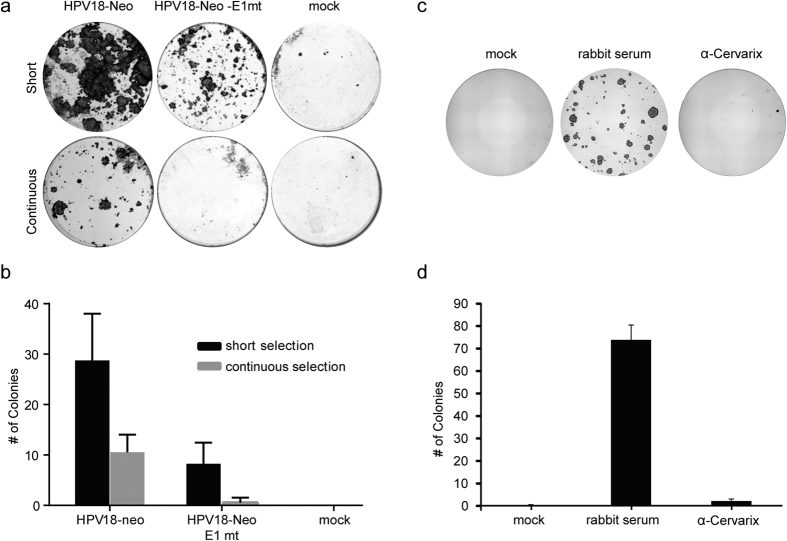
Recombinant HPV18 neo quasiviruses establish long-term infections. (**a**) Primary HFKs were infected with HPV18-Neo containing quasivirions (WT and E1mt) at a VGE/cell of 100 and selected with 50 μg/ml G418 selection for either seven days (short) or continuously until staining. After 16 days feeders were removed and keratinocyte colonies were stained with methylene blue. (**b**) Quantitation of colonies formed in panel (**a**) error bars show SEM (n = 3). (**c**) Primary foreskin keratinocytes were infected with HPV18-Neo containing quasivirions. 48 hours post infection, cells were selected with 200 μg/ml G418 for an additional 96 hours after which selective pressure was removed. Keratinocyte colonies were stained with methylene blue. Infections were done in the presence of serum from rabbits vaccinated with the HPV vaccine Cervarix™ (or pre-immune control). Keratinocyte plates are a representative image. (**d**) Quantitation of colonies formed in panel (**c**) error bars show SEM (n = 3).

In a natural infection with HPV, it is likely that individual cells become infected with very few viral DNA copies. Much larger quantities of DNA are introduced into cells by transfection and this does not mimic natural infection. However, recent advances in HPV biology have made it possible to generate quasiviruses in which the viral genome is encapsidated by expression of L1 and L2^9^, thereby mimicking infectious particles. To generate quasiviruses containing HPV18 marker genomes, 293TT producer cell lines were transfected with the recircularized HPV18-Neo genome together with the L1/L2 containing plasmid, and the resulting quasivirus was purified by density gradient ultracentrifugation. The recombinant virus was used to infect primary HFKs, and following infection, cells were selected with G418, as described in Fig. 4. Using a short-term selection scheme, the HPV18-Neo genome-infected cells formed robust colonies. However, some colonies also developed after infection with a non-replicating genome (E1mt) indicating that, while short term selection can measure infectivity (compare to mock infection), four days of selection is not sufficient to ensure that colony formation is also dependent on the establishment of a replicating viral genome (Fig. 4a and b). In contrast, after continuous selection, very few colonies formed on the E1mt control plates, while infection with wild type HPV18-Neo virus led to robust colony formation (Fig. 4a and b), confirming a key role for viral replication in genome establishment. Furthermore, cell lines established from these continuously selected colonies contained replicating extrachromosomal HPV18-neo genomes (unpublished studies). Therefore, while long-term selection is required to observe the effects of viral replication, short term selected colonies also provide evidence that cells were successfully infected with the recombinant quasivirus. To further confirm that the marker genome-containing quasiviruses infect cells through established viral capsid-mediated entry pathways, we demonstrated that colony formation was successfully abrogated in viral infections where viral particles were first neutralized with serum from rabbits vaccinated with the HPV vaccine, Cervarix™ (Fig. 4c and d).

Continuous selection decreased the number of colonies observed following infection with HPV18-Neo virus (30 vs. 10 colonies; Fig. 4b). Close microscopic inspection indicated that only a subset of colonies (especially after short term selection) contained proliferative cells, as has been described previously^5^,^6^. Proliferative keratinocytes are small, with a cobblestone-like appearance, while non-proliferative cells are flat, with an enlarged cytoplasm and are heterogeneous in shape (see ref. 20 for examples). Therefore, our assay can determine three distinct steps in the infectious process that give rise to persistent infection. The neutralization assay shows that all colonies are due to capsid-mediated delivery of the HPV18-neo genome. However, even in the absence of replication, transient colonies can form that depend on transcription from the delivered viral genome. And finally, only a subset of colonies that contain a transcriptionally active, replicating genomes give rise to a stable proliferative colonies, similar to the infrequent establishment of Epstein-Barr virus derived replicons^21^.

Replication-competent, recombinant papillomavirus genomes are becoming valuable tools for basic research. In a recent elegant study, Leiprecht and colleagues showed that a recombinant SfPV1 (Sylvilagus floridanus Papillomavirus 1, also known as Cotton tail rabbit papillomavirus) viral genome (engineered to express shRNA from the late region) could knock-down cellular targets in the context of viral infection in a rabbit^22^. In addition, HPV18 recombinant genomes expressing luciferase have been used to study the role of APOBEC proteins during viral infection^8^.

In summary, this study describes and characterizes HPV18 genomes that encode selectable markers. The presence of these marker promotes efficient selection of cells containing viral DNA, leading to a robust and quantitative colony forming assay. Although it is not possible to study late events of infection because of disruption of the viral capsid proteins, the genomes can emulate many steps in the viral life cycle. Most notably, quasiviruses carrying these recombinant genomes can initiate long-term infection of primary HFKs. Overall, we believe that these, and similar recombinant genomes can be used to quantitatively study many aspects of the viral life cycle under physiologically relevant conditions.

## Methods

### Plasmids

The wild-type HPV18 genome cloned into pBR322 was described previously^23^. HPV18 genomes with a translation termination linker in the E1 ORF (with the suffix E1mt) were generated by inserting a short DNA fragment containing stop codons in all open reading frames into the HpaI site (nt 2472) of the viral genome. The pCpG-Neo vector is based on pCpG free-vitroNmcs (Invivogen; cat. no. pcpgvtn-mcsg2). First, a fragment containing the bacterial R6K origin of replication and the CpG-free Neomycin resistance cassette was PCR amplified from the parental plasmid, followed by restriction digest with PacI and HpaI. The new plasmid (pCpG-Neo) is 2198 bp in length and expresses the neomycin resistance cassette from the SV40 promoter/enhancer, using the beta globin polyA site, and also from the prokaryotic I-EC2K promoter. To create the recombinant HPV18-Neo genome, the Neomycin cassette was PCR amplified from pCpG free-vitroNmcs, incorporating flanking BsiWI restriction sites. The PCR amplicon was cloned into Asp718 restriction sites within the HPV18 genome. This replaces a 1479 bp fragment (nt. 4795-6273 in HPV18) with the Neomycin cassette.

To generate an HPV18-GFP virus, a CpG-free GFP cassette (not used in this study) similar to the Neomycin cassette was synthesized by GeneArt. The GFP cassette consists of the SV40 enhancer/promoter, a CpG free GFP gene (based on the pSELECT-CGFP; Invivogen; cat. no. psetz-zgfpsh), and the beta-globin poly-A signal. To facilitate later transgene cloning, the GFP coding sequence was flanked by BglII and BclI restriction sites. This cassette was cloned into HPV18 at the same (Asp718) position as described for the Neomycin cassette, thus creating the HPV18-GFP virus.

To generate HPV18 genomes containing a GFP-Zeocin cassette, the GFP-Zeocin fusion gene was PCR amplified from pSELECT-CGFP (Invivogen; cat. no. psetz-zgfpsh) incorporating flanking BglII and BclI restriction sites. The fusion gene was cloned into the corresponding sites of the HPV18-GFP genome, effectively replacing GFP by the GFP-Zeocin fusion, thereby creating HPV18-GFP::SH recombinant genome. PCR primers are listed in supplementary Table 1.

DNA standards used in standard curves for qRT-PCR of spliced HPV18 transcripts E1^E4 and E6*I [nt 914 to 929 and 3434 to 3684, and E6 nt 105 to 233 and 416 to 929 respectively), were described previously^9^. pRSV-neo was purchased from the American Type Culture Collection (ATCC 37198). The β-actin plasmid was obtained from Addgene (#27124^24^). pDRIVE-TBP, a plasmid containing the TATA box binding protein (TBP) cDNA was purchased from Open Biosystems (cat. no. MHS6278-202802567).

### Cell culture

Human neonatal foreskin specimens were collected with informed consent of parents, or guardians, and with approval from the NIH Institutional Review Board. Primary human keratinocytes were isolated from neonatal foreskins as described previously^20^, Cells were expanded in Rheinwald-Green F medium (3:1 Ham’s F-12/high-glucose Dulbecco’s modified Eagle’s medium [DMEM], 5% fetal bovine serum, 0.4 μg/ml hydrocortisone, 8.4 ng/ml cholera toxin, 10 ng/ml epidermal growth factor, 24 μg/ml adenine, 6 μg/ml insulin) on a layer of lethally irradiated J2/3T3 murine fibroblasts. Antibiotics were not used unless otherwise noted. For experiments using G418 selection, HFKs were cultured on G418-resistant NIH-3T3 J2 murine fibroblasts^9^.

### Electroporation of keratinocytes with viral DNA

Viral genomes (and derivatives) were cleaved from their prokaryotic vectors with EcoRI, and recircularized at a concentration of 5 μg/ml DNA by overnight incubation with T4 DNA ligase^3^. One million human foreskin keratinocytes were electroporated with 1 μg recircularized HPV18 DNA and 1 μg selectable marker/control DNA using the Amaxa Human Keratinocyte Nucleofector kit (program setting T007).

### Quantitative immortalization assay

5 × 10^5^ electroporated cells were plated onto G418-resistant resistant NIH-3T3 J2 fibroblasts. After 24 h, electroporated keratinocytes were selected for the indicated amount of time. HFKs were cultured in the presence of irradiated J2-3T3 cells, until distinct keratinocyte colonies were visible. Colonies were fixed with formalin and stained with methylene blue.

### RNA extraction and qRT-PCR detection of viral transcripts

Total RNA was isolated with the RNeasy Mini-RNA extraction kit (Qiagen). RNA quality was assessed using a RNA 6000 Nano Kit (Bioanalyzer; Agilent Technologies). RNA integrity numbers (RIN) for all samples were greater than 9. One μg of RNA was reverse transcribed using the Transcriptor First-Strand Synthesis kit (Roche). Real-time qRT-PCR was performed with the ABI Prism 7900HT Sequence Detector (Applied Biosystems) and SYBR green PCR master mix (Roche). Each reaction mixture contained 10 μl SYBR green master mix, cDNA from the equivalent of 100 ng RNA, and 150 nM each oligonucleotide primer in a total volume of 20 μl. In each run, a 10-fold dilution series (1 × 10^5^ to 1 × 10^−1^ fg) of pUC57-E1^E4, pUC57-E6*I, or pDRIVE-TBP was included to generate a standard curve of cycle threshold versus log10 quantity. PCR was performed in triplicate at 95 °C for 15 min, followed by 40 cycles of denaturation at 95 °C for 10 s and annealing and extension at 60 °C for 30 s. For the sequences of the primers used, see supplementary Table 1.

### DNA qPCR for viral genome copy number

Total cellular DNA was isolated from keratinocytes with the DNeasy Blood and Tissue kit (Qiagen) at 96 hours post-electroporation. DNA samples were digested with DpnI and NcoI restriction endonucleases. After digestion, an aliquot was analyzed by qPCR using 150 nM each primer and SYBR green master mix (Roche). The reaction conditions consisted of a 15-min 95 °C activation cycle, 40 cycles of 10 s 95 °C denaturation and 30 s 60 °C annealing, followed by a final elongation. Standard curves for HPV18 and human β-actin were included. For the sequences of the primers used, see supplementary Table 1.

### Southern blot analysis

Total DNA was harvested with the DNeasy Blood and Tissue kit (Qiagen). For transient DNA replication analysis DNA was harvested 96 hours post electroporation. Two μg total DNA was digested with NcoI and DpnI. These enzymes linearize the HPV18 genome to unit length and remove any unreplicated viral DNA, respectively. DNA harvested from established colonies was not digested. After digestion, samples were separated on 1% Tris-acetate-EDTA (TAE) agarose gels. DNA was visualized with 0.5 mg/ml ethidium bromide and transferred onto nylon membranes with a TurboBlotter downward transfer system (Whatman). Membranes were UV cross-linked, dried, and incubated overnight with hybridization buffer (3X SSC, 2% SDS, 5xDenhardt’s solution, 0.2 mg/ml sonicated salmon sperm containing 25 ng [^32^P]-dCTP-labeled HPV18 DNA). Radiolabeled probe was generated from 50 ng of gel purified linear HPV18 DNA using the Random Prime DNA labeling kit (Roche). Hybridized DNA was visualized by phosphorimaging on a Typhoon Scanner (GE Bioscience).

### Preparation of HPV18 quasiviruses

HPV18 “quasivirions” were made as previously described^7^,^9^. 7 × 10^6^ 293TT cells were transfected with 19 μg recircularized HPV18 marker genome and 19 μg of pSheLL16 L1/L2 packaging plasmids. Cells were lysed in buffer containing 0.5% Triton X-100 (Sigma), 0.1% Benzonase (EMD Millipore), 0.1% Plasmid Safe (Epicenter) and 25 mM ammonium sulfate (pH 9). Following a virion maturation step (overnight incubation at 37 °C), virions were purified by ultracentrifugation through a three-step (27%, 33%, and 39%) OptiPrep (Sigma) gradient. Gradient fractions were screened for HPV18 DNA by qPCR (for the sequences of the primers used, see supplementary Table 1) and for L1 capsid protein content by SDS-PAGE. Fractions containing both viral genomes and L1 protein were pooled and used as viral stocks. Viral genome equivalents (VGEs) were calculated by extracting viral DNA from capsids in 100 μl digestion solution (20 mM Tris, pH 8, 20 mM DTT, 20 mM EDTA, 0.5% SDS and 0.2% proteinase K), incubated at 50 °C for 20 minutes. DNA was purified with a Qiaquick PCR purification kit (Qiagen), and quantified by qPCR using the standard curve method. Generation of recombinant papillomaviruses, and their packaging in quasivirus, was approved by NIH Division of Occupational Health and Safety (DOHS), Registration number RD-12-X-14. All methods were performed in accordance with the relevant DOHS guidelines and regulations.

### Indirect immunofluorescence

Cells cultured on glass coverslips were fixed with 4% paraformaldehyde, permeabilized with 0.1% Triton X-100 (Sigma) in phosphate-buffered saline (PBS), and blocked in 0.25% bovine serum albumin (BSA)-gelatin in PBS. The cells were stained with a primary anti-GFP antibody (Abcam, ab1218) for 1 h at 37 °C. Fluorescent secondary antibodies labeled with Alexa 488 (Jackson Immunoresearch) were used at a 1:50 dilution. Coverslips were mounted by using ProLong Gold with 4′,6-diamidino-2-phenylindole (DAPI; Molecular Probes). Cells were visualized with a Leica SP5 confocal microscope.

### Infection with HPV18-Neo quasivirus

Primary human foreskin keratinocytes (HKFs; passage 2 to 5 post-isolation) were seeded at 1-3 × 10^5^ cells per 10 cm plate containing 1 × 10^6^ lethally irradiated G418-resistant NIH 3T3 J2 fibroblasts. Forty-eight hours later, cells were infected with a HPV18-Neo based quasivirus at 100 viral genome equivalents (VGE)/cell. Where indicated, the virus was pre-incubated with a 1:450 dilution of sera from rabbits vaccinated with Cervarix™ (or a pre-immune serum control) for one hour on ice (kind gift from Drs. Schiller and Day, NCI^25^). Inoculum was added to 4 ml of F-media per plate of cells and allowed to adsorb for one hour at 4 °C. Six ml of F-media was added and the plate moved to 37 °C. Forty-eight hours after infection, cells were selected for 96 additional hours in 200 μg/ml G418. Finally, cells were incubated for an additional 2–3 weeks in the absence of selection media. To show the ability to support long term replication, infected cells were continuously selected with 50 μg/ml G418.

### Time lapse live cell imaging

Transfected human foreskin keratinocytes were plated on irradiated J2–3T3 feeders in 6-well culture dishes in F-medium, and were imaged for up to 31 days by Phase Contrast and GFP fluorescence using an Incucyte incubator microscope (Essen Bioscience, Ann Arbor, MI, USA). Images and movies (supplementary materials) were created using the Incucyte software.

## Additional Information

**How to cite this article**: Van Doorslaer, K. *et al*. Novel recombinant papillomavirus genomes expressing selectable genes. *Sci. Rep*. **6**, 37782; doi: 10.1038/srep37782 (2016).

**Publisher's note:** Springer Nature remains neutral with regard to jurisdictional claims in published maps and institutional affiliations.

## Supplementary Material

Supplementary Information

Supplementary Movie S1

## References

[b1] Mesri E. A., Feitelson M. A. & Munger K. Human viral oncogenesis: a cancer hallmarks analysis. Cell host & microbe 15, 266–282, doi: 10.1016/j.chom.2014.02.011 (2014). 24629334 PMC3992243

[b2] LaPorta R. F. & Taichman L. B. Human papilloma viral DNA replicates as a stable episome in cultured epidermal keratinocytes. Proc. Natl. Acad. Sci. USA 79, 3393–3397 (1982). 6285348 10.1073/pnas.79.11.3393 PMC346426

[b3] McCance D. J., Kopan R., Fuchs E. & Laimins L. A. Human papillomavirus type 16 alters human epithelial cell differentiation *in vitro*. Proc. Natl. Acad. Sci. USA 85, 7169–7173 (1988). 2459699 10.1073/pnas.85.19.7169 PMC282145

[b4] Meyers C., Mayer T. J. & Ozbun M. A. Synthesis of infectious human papillomavirus type 18 in differentiating epithelium transfected with viral DNA. J Virol 71, 7381–7386 (1997). 9311816 10.1128/jvi.71.10.7381-7386.1997 PMC192083

[b5] Lace M. J. . Human papillomavirus (HPV) type 18 induces extended growth in primary human cervical, tonsillar, or foreskin keratinocytes more effectively than other high-risk mucosal HPVs. J Virol 83, 11784–11794, doi: 10.1128/JVI.01370-09 (2009). 19740985 PMC2772696

[b6] Lace M. J., Turek L. P., Anson J. R. & Haugen T. H. Analyzing the Human Papillomavirus (HPV) Life Cycle in Primary Keratinocytes with a Quantitative Colony-Forming Assay. Current protocols in microbiology 33, 14B 12 11–13, doi: 10.1002/9780471729259.mc14b02s33 (2014). 24789595

[b7] Pyeon D., Lambert P. F. & Ahlquist P. Production of infectious human papillomavirus independently of viral replication and epithelial cell differentiation. Proc Natl Acad Sci USA 102, 9311–9316, doi: 10.1073/pnas.0504020102 (2005). 15958530 PMC1166641

[b8] Warren C. J. . APOBEC3A functions as a restriction factor of human papillomavirus. J Virol 89, 688–702, doi: 10.1128/JVI.02383-14 (2015). 25355878 PMC4301161

[b9] Stepp W. H., Meyers J. M. & McBride A. A. Sp100 provides intrinsic immunity against human papillomavirus infection. mBio 4, e00845–00813, doi: 10.1128/mBio.00845-13 (2013). 24194542 PMC3892783

[b10] McKinney C., Kim M. J., Chen D. & McBride A. A. Brd4 activates early HPV18 transcription upon quasivirus infection. doi: 10.1128/mBio.01644-16 (2016). PMC5120138 27879331

[b11] Van Doorslaer K., Chapman S., Khan J. & McBride A. A. Viral transcriptional enhancer is required for optimal persistence of an oncogenic papillomavirus genome. in preparation (2016). 10.1128/mBio.01758-17 PMC5698554 29162712

[b12] Takahashi Y., Nishikawa M. & Takakura Y. Development of safe and effective nonviral gene therapy by eliminating CpG motifs from plasmid DNA vector. Front Biosci (Schol Ed) 4, 133–141 (2012). 22202048 10.2741/256

[b13] Tolmachov O. E. Building mosaics of therapeutic plasmid gene vectors. Current gene therapy 11, 466–478 (2011). 22023476 10.2174/156652311798192798

[b14] Hodges B. L., Taylor K. M., Joseph M. F., Bourgeois S. A. & Scheule R. K. Long-term transgene expression from plasmid DNA gene therapy vectors is negatively affected by CpG dinucleotides. Molecular Therapy 10, 269–278 (2004). 15294174 10.1016/j.ymthe.2004.04.018

[b15] Hyde S. C. . CpG-free plasmids confer reduced inflammation and sustained pulmonary gene expression. Nature biotechnology 26, 549–551 (2008). 10.1038/nbt1399 18438402

[b16] Warren C. J., Van Doorslaer K., Pandey A., Espinosa J. M. & Pyeon D. Role of the host restriction factor APOBEC3 on papillomavirus evolution. Virus Evolution 1, vev015 (2015). 27570633 10.1093/ve/vev015 PMC4999249

[b17] Shackelton L. A., Parrish C. R. & Holmes E. C. Evolutionary basis of codon usage and nucleotide composition bias in vertebrate DNA viruses. Journal of molecular evolution 62, 551–563 (2006). 16557338 10.1007/s00239-005-0221-1

[b18] Yew N. S., Przybylska M., Ziegler R. J., Liu D. & Cheng S. H. High and sustained transgene expression *in vivo* from plasmid vectors containing a hybrid ubiquitin promoter. Molecular therapy: the journal of the American Society of Gene Therapy 4, 75–82, doi: 10.1006/mthe.2001.0415 (2001). 11472109

[b19] Lanza A. M., Kim D. S. & Alper H. S. Evaluating the influence of selection markers on obtaining selected pools and stable cell lines in human cells. Biotechnology journal 8, 811–821 (2013). 23450727 10.1002/biot.201200364

[b20] Chapman S., Liu X., Meyers C., Schlegel R. & McBride A. A. Human keratinocytes are efficiently immortalized by a Rho kinase inhibitor. The Journal of clinical investigation 120, 2619–2626, doi: 10.1172/JCI42297 (2010). 20516646 PMC2898606

[b21] Leight E. R. & Sugden B. Establishment of an oriP replicon is dependent upon an infrequent, epigenetic event. Mol Cell Biol 21, 4149–4161 (2001). 11390644 10.1128/MCB.21.13.4149-4161.2001 PMC87076

[b22] Leiprecht N. . A novel recombinant papillomavirus genome enabling *in vivo* RNA interference reveals that YB-1, which interacts with the viral regulatory protein E2, is required for CRPV-induced tumor formation *in vivo*. American journal of cancer research 4, 222 (2014). 24959377 PMC4065403

[b23] Romanczuk H. & Howley P. M. Disruption of either the E1 or the E2 regulatory gene of human papillomavirus type 16 increases viral immortalization capacity. Proc. Natl. Acad. Sci. USA 89, 3159–3163 (1992). 1313584 10.1073/pnas.89.7.3159 PMC48824

[b24] Rodriguez A. J., Shenoy S. M., Singer R. H. & Condeelis J. Visualization of mRNA translation in living cells. J Cell Biol 175, 67–76, doi: 10.1083/jcb.200512137 (2006). 17030983 PMC2064499

[b25] Day P. M. . *In vivo* mechanisms of vaccine-induced protection against HPV infection. Cell host & microbe 8, 260–270, doi: 10.1016/j.chom.2010.08.003 (2010). 20833377 PMC2939057

