# Supplementary material for: Novel recombinant papillomavirus genomes expressing selectable genes
Source: Sci Rep. 2016 Nov 28;6:37782. doi: 10.1038/srep37782 (PMC5125267; doi:10.1038/srep37782)
Supplement: Supplementary Information [file srep37782-s1.pdf]

Supplemental Material

Novel recombinant papillomavirus genomes expressing selectable genes

Koenraad Van Doorslaer, Samuel Porter, Caleb McKinney, Wesley H. Stepp, and Alison A. McBride

Supplemental Table 1

Sequence of primers used in this study.

**lab ID**	**sequence (5' - 3')***	**Restriction enzyme recognition sites**	**position in HPV18 genome (5' - 3')**	**Purpose**
AM2257	GGCC <u>GGATCC</u> <u>GCTAGC</u> TAAAATACAGCATAGCAAAA	BamHI and NheI	N/A	Construction of pCPG-Neo
AM2258	CCGG <u>GGATCC</u> <u>TGTACA</u> AATCAGCAGTTCAACCTGTT	BamHI and BsrGI	N/A	Construction of pCPG-Neo
AM2282	CCGG <u>CGTACG</u> TAAAATACAGCATAGCAAAA	BsiWI	N/A	Construction of HPV18-Neo
AM2283	CCGG <u>CGTACG</u> GGCCTGAAATAACCTCTGAA	BsiWI	N/A	Construction of HPV18-Neo
AM2762	ACTG <u>AGATCT</u> CCGGTCACCATGGTTTCTAA	BglII	N/A	Construction of HPV18-GFP::SH
AM2763	ACTG <u>TGATCA</u> TTAGTCCTGCTCCTCTGCCA	BclI	N/A	Construction of HPV18-GFP::SH
AM3210	CAAGACAGTATTGGAACTTACAGAGGTG	N/A	208 - 408	Quantify HPV18 E6*1 (crosses splice site)
AM3211	CTGGCCTCTATAGTGCCCAGC	N/A	515 - 495	Quantify HPV18 E6*1 (crosses splice site)
AM3212	CAACAATGGCTGATCCAGAAGTAC	N/A	907 - 3436	Quantify HPV18 E1^E4 (crosses splice site)
AM3213	TAGGTCTTTGCGGTGCCC	N/A	3535 - 3518	Quantify HPV18 E1^E4 (crosses splice site)
AM3220	CACAATACTATGGCGCGCTTT	N/A	96 - 116	Quantify HPV18 DNA following transfection (across DpnI site)
AM3221	CCGTGCACAGATCAGGTAGCT	N/A	162 - 142	Quantify HPV18 DNA following transfection (across DpnI site)

* Restriction enzyme recognition sites are underlined

Supplemental Movie 1

Time lapse live cell imaging

Human foreskin keratinocytes, transfected with HPV18-GFP::SH, were plated on irradiated J2-3T3 feeders in 6-well culture dishes in F-medium, and were imaged for up to 31 days while cells were undergoing zeocin selection. GFP fluorescence was detected using an Incucyte incubator microscope (Essen Bioscience, Ann Arbor, MI, USA). The movie was created using the Incucyte software, Incucyte ZOOM 2015.
